# AgingPLUS: A Randomized Trial to Increase Physical Activity in Middle-Aged and Older Adults

**DOI:** 10.1093/geroni/igab046.1680

**Published:** 2021-12-17

**Authors:** Manfred Diehl, Jennifer Schrack

## Abstract

Engagement in physical activity (PA) has well-documented benefits for delaying or preventing age-related diseases. Thus, it is important to study innovative ways to increase PA in the adult population. This symposium describes AgingPLUS, an ongoing trial that addresses three psychological mechanisms to increase adults’ PA: Negative views of aging (NVOA), low self-efficacy beliefs, and deficient goal-planning skills. The symposium also presents preliminary findings, based on a pre-pandemic subsample, on changes in explicit NVOA, implicit VOA, and changes in PA. Diehl et al. describe the theoretical background and study design of the ongoing RCT. This also includes the main study hypotheses. Rebok et al. present preliminary findings showing significant effects of the intervention on NVOA and frequency of moderate intensity exercise. Effects on physical function and accelerometry measures were not statistically significant in this subsample. Tseng et al. examined the effects of the intervention on two measures of implicit VOA: a lexical decision-making task (LDMT) and the Brief Implicit Association Test (BIAT). Findings showed that differences in post-intervention latencies on the LDMT were not statistically significant. Differences on post-intervention BIAT d scores also failed to be significant. Finally, Nehrkorn-Bailey et al. tested a multiple mediator model examining the mediational role of self-efficacy (SE) and exercise intention (EI) on PA. Results showed that Week 4 SE significantly mediated the effect of intervention condition to Week 8 anticipated PA engagement. Week 4 EI significantly mediated the effect of intervention condition on Month 6 PA engagement. Anticipated PA effects predicted subsequent involvement in PA.

